# Supplementary material for: Towards unified AI-driven fracture mechanics: the extended deep energy method (XDEM)
Source: Nat Commun. 2026 Aug 15;17:8492. doi: 10.1038/s41467-026-76748-1 (PMC13484471; doi:10.1038/s41467-026-76748-1)
Supplement: Supplementary file 3 [file 41467_2026_76748_MOESM3_ESM.zip › pic/Results/Stress_intensity_factor/Mode1/crack_gamma_embedding.pdf]

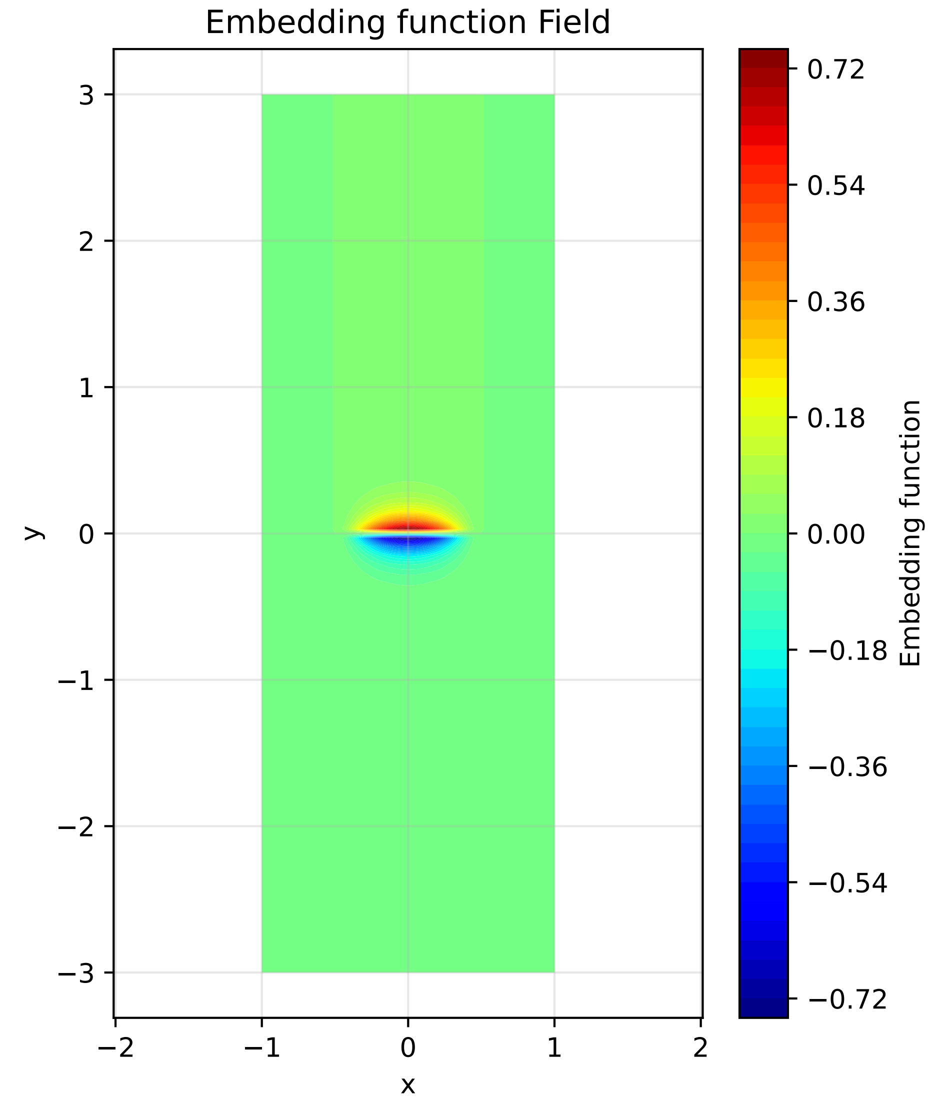

Embedding function Field
y
x
3
2
1
0
−1
−2
−3
−2
−1
0
1
2
0.72
0.54
0.36
0.18
0.00
−0.18
−0.36
−0.54
−0.72
Embedding function
